# A Bayesian Method for Evaluating and Discovering Disease Loci Associations

**DOI:** 10.1371/journal.pone.0022075

**Published:** 2011-08-10

**Authors:** Xia Jiang, M. Michael Barmada, Gregory F. Cooper, Michael J. Becich

**Affiliations:** 1 Department of Biomedical Informatics, University of Pittsburgh, Pittsburgh, Pennsylvania, United States of America; 2 Department of Human Genetics, University of Pittsburgh, Pittsburgh, Pennsylvania, United States of America; 3 Intelligent Systems Program, University of Pittsburgh, Pittsburgh, Pennsylvania, United States of America; Dana-Farber Cancer Institute, United States of America

## Abstract

**Background:**

A genome-wide association study (GWAS) typically involves examining representative SNPs in individuals from some population. A GWAS data set can concern a million SNPs and may soon concern billions. Researchers investigate the association of each SNP individually with a disease, and it is becoming increasingly commonplace to also analyze multi-SNP associations. Techniques for handling so many hypotheses include the Bonferroni correction and recently developed Bayesian methods. These methods can encounter problems. Most importantly, they are not applicable to a complex multi-locus hypothesis which has several competing hypotheses rather than only a null hypothesis. A method that computes the posterior probability of complex hypotheses is a pressing need.

**Methodology/Findings:**

We introduce the Bayesian network posterior probability (BNPP) method which addresses the difficulties. The method represents the relationship between a disease and SNPs using a directed acyclic graph (DAG) model, and computes the likelihood of such models using a Bayesian network scoring criterion. The posterior probability of a hypothesis is computed based on the likelihoods of all competing hypotheses. The BNPP can not only be used to evaluate a hypothesis that has previously been discovered or suspected, but also to discover new disease loci associations. The results of experiments using simulated and real data sets are presented. Our results concerning simulated data sets indicate that the BNPP exhibits both better evaluation and discovery performance than does a *p*-value based method. For the real data sets, previous findings in the literature are confirmed and additional findings are found.

**Conclusions/Significance:**

We conclude that the BNPP resolves a pressing problem by providing a way to compute the posterior probability of complex multi-locus hypotheses. A researcher can use the BNPP to determine the expected utility of investigating a hypothesis further. Furthermore, we conclude that the BNPP is a promising method for discovering disease loci associations.

## Introduction

The advent of high-throughput technologies has enabled genome-wide association studies (GWAS). A GWAS can involve examining a million representative single-nucleotide polymorphisms (SNPs) in individuals from some population. Often GWAS are conducted on cases and controls, where cases are individuals with a disease and controls are individuals without the disease. We then investigate the statistical association of each SNP with the disease. In doing so, a million hypotheses (disease-SNP relationships) or more may be investigated.

GWA studies provide researchers unprecedented opportunities to investigate the complex genetic basis of diseases such as cancer. For example, GWAS have indicated that alleles in the *FGFR2* gene are associated with sporadic postmenopausal breast cancer [1]; that five loci are associated with breast cancer including the plausible causative genes *FGFR2*, *TNRC9*, *MAP3K1*, and *LSP1* [2]; and that *GAB2* alleles may modify Alzheimer's risk in *APOE* 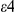 carriers [3]. Studies investigating SNPs in tumorous and non-tumorous tissue have revealed somatic mutations possibly associated with cancer. For example, recent studies showed eight genes somatically mutated in glioblastoma tumors [4], and 26 genes somatically mutated in lung adenocarcinoma [5]. The 1000 Genomes Project plans to produce sequence coverage that will extend the list of human genetic variation [6], and gene-environment-wide association studies are emerging [7], both of which will increase the number of hypotheses investigated. *Epistasis* is the interaction between two or more genes to affect a phenotype such as disease susceptibility. Biologically, epistasis likely arises from physical interactions occurring at the molecular level. Statistically, epistasis refers to an interaction between multiple loci such that the net affect on phenotype cannot be predicted by simply combining the effects of the individual loci. Researchers now believe that epistasis may account for a significant portion of the dark matter of genetic risk for disease [8], and it is becoming increasingly commonplace for researchers to investigate epistasis using GWAS data sets [9], [10], which dramatically increases the number of hypotheses investigated. For example, if we only considered all 2-SNP interactions when there are 500,000 SNPs, we would have 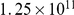 additional hypotheses.

These exciting possibilities for learning potential disease risk from high-dimensional data sets presents us with a challenge - namely how do we analyze and interpret our results when there are possibly billions of hypotheses? The hypothesis testing involved here is substantially different than that involved in a typical analysis where we might analyze the effect of a new drug. In this latter case, we are analyzing only one hypothesis, and the drug has a fairly high prior probability of being effective, otherwise the study would not have been considered. In discovery studies involving many hypotheses, each hypothesis has a very low prior probability.

Historically, the most common strategy for handling this multiple hypotheses testing problem has been to control type I error (false discovery) by using the Bonferroni correction to constrain the family-wise error rate. For example, the results in [3] were reported as being significant with Bonferroni correction. However, these corrected results often fail to duplicate across studies [8]. More recently developed techniques include the false discovery rate [11], false positive report probability [12], and Bayesian false discovery probability [13].

These methods all have the same purpose, namely to provide us with a way to decide which SNPs to “flag as noteworthy for further investigation” [13]. A difficulty with these methods is that they are not applicable to a complex multi-locus hypothesis, which has several competing hypotheses rather than only a null hypothesis. However, as mentioned above, it is becoming increasingly commonplace to investigate gene-gene interactions. So, a method that computes the posterior probability of a complex multi-locus hypothesis (and thereby flags the SNPs in the hypothesis as noteworthy) is a pressing need. In the Methods section we present a fully Bayesian method called the *Bayesian network posterior probability* (BNPP) method that is able to handle multi-locus hypotheses by computing the posterior probability of a hypothesis; it does so by assigning prior probabilities over all the hypotheses and computing the likelihoods of specialized Bayesian network structures [14], as explained below. The Results section shows results of experiments illustrating the effectiveness of the BNPP at both evaluation and discovery, using both simulated and real data sets. In the remainder of this section we briefly review current methods and point out difficulties that they encounter.

When testing multiple hypotheses as in a GWAS, one of the hypotheses is likely to have a significant *p*-value by chance. As a result, researchers often use the *Bonferroni correction* to control the family-wise error rate by multiplying the *p*-value by the number of hypotheses *n*. For example, if 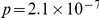 for a given SNP-outcome association and 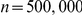, then the Bonferroni-corrected *p*-value is 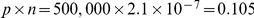. This result would not be deemed significant by most standards, and the null hypothesis would not be rejected. A related correction is the *Šidák correction*, which is 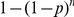.

Wakefield [13] notes that in the case of a GWAS the Bonferroni correction will often be an overly conservative procedure since at least in the current early stages of such studies we are more concerned with avoiding missed associations, and making some false discoveries is not too high a cost to pay to find real associations. Neapolitan [15] has a more fundamental problem with the Bonferroni correction. He argues that it is a misguided practice, and that the significance we attach to a result concerning a particular hypothesis cannot depend on the number of hypotheses we happen to test along with that hypothesis.

Regardless of one's stance on this matter, there are clear difficulties in applying the Bonferroni correction in GWA studies. Suppose that one study investigates 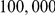 SNPs while another investigates 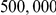 SNPs. Suppose further that the data concerning a particular SNP and the disease is identical in the two studies. Due to the different corrections, that SNP could be reported as significant in one study but not the other. Yet the data concerning the SNP is identical in the two studies. As noted earlier, in GWAS results are often not duplicated across studies. One reason may be the practice of using different corrections across different studies. Initially GWAS data were analyzed by investigating only 1-SNP models (hypotheses). We use the terms “model” and “hypothesis” interchangeably. Strictly speaking, the hypothesis is the statement that the model is correct.

So if there were 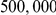 SNPs, there would be 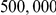 hypotheses. Based on these studies, quite a few results have been reported as significant with correction [3], [16]–[18]. It is becoming increasingly popular to also investigate 2-SNP models in the effort to identify epistatic relationships [8]–[10]. As mentioned above, if there are 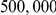 SNPs, there are about 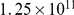 2-SNP models. If the researchers who previously reported significant results had also investigated the 2-SNP models, the corrections would have been based on many more hypotheses and the results likely would not have been reported as significant.

Realizing these problems, some researchers [17], [19] have suggested that we uniformly use a Bonferroni correction assuming 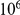 independent tests in GWA studies. This value was arrived at based on assuming only 1-SNP models are tested. If this were done, the problem concerning using different corrections when analyzing 1-SNP models in different studies would be addressed, but the problem of analyzing 1-SNP models along with 2-SNP models would not. We could correct the significance of a 1-SNP model assuming 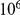 tests, and then perhaps we could correct the significance of a 2-SNP model assuming a much larger number of tests. However, even if we did all this we would still have the problem identified by Wakefield [13] concerning the correction being overly conservative, and the problem that we have ignored the probability of the data given the alternate hypothesis (power).

Benjamin and Hochberg [11] concluded that a desirable error rate to control is the expected proportion of errors among the rejected null hypotheses, which they termed the *false discovery rate* (FDR). That is, the FDR is *E*(*V*/*R*), where *E* denotes expected value, 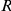 is the number of null hypotheses rejected and 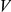 is the number of true null hypotheses rejected (Recall that we make a discovery when we reject a null hypothesis). They prove the following theorem, which enables us to control the FDR in practice: Suppose we have 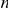 hypotheses with corresponding 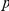-values 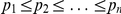. Denote by 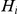 the null hypothesis corresponding to 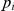. Let
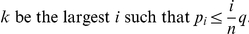
Then if we reject 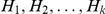, the FDR is ≤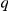.

Storey [20] gave the FDR a Bayesian interpretation, showed that the *E*(*V*/*R*|*R*>0) (called the *positive FDR*) does not depend on *n*, and defined the *q*-value. Storey and Tibshirani [21] develop an empirical method for estimating the *q*-value from the observed distribution of *p*-values.

Storey et al. [22] developed a method that computes the posterior probability that a locus is in the true model given the data, without ever estimating the entire true model. However, this method is applicable to the situation in which we are investigating many phenotype traits simultaneously rather than a single trait. The FDR was used to correct for multiple comparisons in this method.

The next two methods discussed concern the following analysis. We test 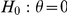 vs. 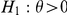 where 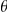 is the log odds ratio. For example, if 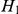 is the hypothesis that a particular SNP 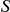 is associated with disease 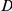,
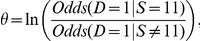
where by *D* = 1 we mean the disease is present, and by 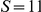 we mean that an individual has two copies of the mutant allele 1. We have assumed in this example that the wild type is dominant. The model assumes a test statistic 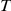 with 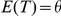. For example, we may fit a logistic regression model so that *T* is the maximum likelihood estimate of the log odds ratio.

The *false positive report probability* (FPRP) [12] is defined as follows:
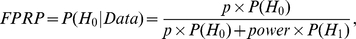
where 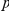 is the 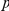-value, the 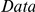 are assumed to be the result that 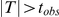 where 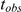 is the observed value of *T*, and 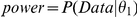 is evaluated at a pre-specified 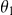.

There are a number of difficulties with this method:

1. Information is being lost by considering the data as being the result that 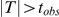 rather than the point value (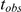) we observed.
2. How do we decide on a particular value of 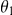
? Perhaps we should consider a range of values.
3. The odds ratio only considers two possibilities, either a condition is present or it is not. However, we may want to model that there could be a different effect on disease for each of the three values a SNP can obtain.
4. We can only consider a null hypothesis 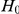 and an alternative hypothesis 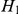. However, if we model 2-SNP, 3-SNP models, etc., there are several competing models (hypotheses) besides the one whose probability we are computing and each has a different likelihood. This issue is discussed in more detail in the Methods section.

As an alternative to the FPRP, Wakefield [13] developed the *Bayesian false discovery probability* (BFDP) which addresses several of the difficulties just presented. We do not go into its details here, but only mention that it does not attend to Difficulties 3 and 4.

A Bayesian method was used to compute the strength of association of a finding obtained using GWAS data in the Wellcome Trust Case Control Consortium study [23]. This method identified the following three hypotheses concerning the association of a single SNP with the disease:

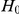
 denotes a model with no association with the disease.

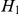
 denotes a two-parameter model with an additive effect on the log-odds scale. That is, the log-odds for the *i*th individual is
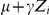
where 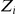 is the genotype (codes as 0, 1, or 2), 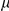 is the baseline odds, and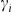 is the increase in odds for every copy of the allele coded as 1.

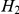
 denotes a three-parameter model with an additive effect on the log-odds scale.

The Bayes factor for 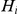 versus 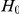 is
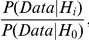
where
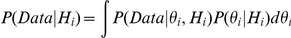
and 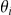 denotes the parameters in the model. For all three models a logistic regression model was used for the likelihood 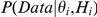. The log of the Bayes factor was reported for both 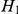 and 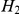. This method addresses Difficulty 3 to some extent by considering three values of the genotype. However, it does not concern multi-locus hypotheses and address Difficulty 4.

Zhang and Liu [24] developed Bayesian epistasis association mapping (BEAM) for the purpose of discovery. However, the method does assign prior probabilities to loci being associated with the disease and reports posterior probabilities. It does not consider multiple competing hypotheses.

Sebastiani et al. [25] computed the posterior probabilities of individual SNPs using a likelihood like the one presented here. These researchers performed a GWAS concerning 298,734 SNPs with the purpose of developing a system for predicting *extended longevity* (EL). In the first stage of their investigation they computed the posterior probability of each SNP being associated with EL, where the prior probability was assumed to be 0.5. They used these posterior probabilities to rank the SNPs and thereby flag SNPs to include in the second phase of the investigation, which was to decide which SNPs to include in the predictive model.

Bayesian networks have previously been used to discover disease loci interactions using likelihoods [10], [26]–[28]. However, we know of no previous research that used them to determine the posterior probability of a complex multi-SNP model being associated with the disease.

## Methods

We developed the BNPP method specifically to enable us to compute the posterior probability of multi-locus models, which addresses Difficulty 4 above; however, it also attends to the other three difficulties.

A 1-locus model is the model that a single locus by itself is associated with a phenotype such as a disease, a 2-locus model is the model that two loci together are associated with a phenotype, and so on. The BNPP method represents such models using particular types of Bayesian network structures and computes the posterior probability of a model based on the likelihoods of these structures and their prior probabilities.

The BNPP was designed for the purpose of *flagging SNPs for further investigation*; that is, it is intended to compute the posterior probability of a model that was already discovered or conjectured. We previously used Bayesian networks for discovery of disease loci associations [10], [26], [27]. However, we only computed the likelihoods of the models; we did not consider their prior probabilities. A bigger model (more loci) will sometimes have a higher likelihood, but be less probable because of its smaller prior probability. The BNPP accounts for this situation, whereas a method that only looks at likelihoods does not. So, the BNPP is also a new, promising technique for *discovery*.

Before describing the BNPP algorithm, we first review Bayesian networks on which the algorithm is based.

### Bayesian Networks

Bayesian networks [14] have been used for modeling and knowledge discovery in many domains, including applications to bioinformatics [29]. A *Bayesian network* (BN) consists of a *directed acyclic graph* (DAG) 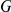
whose set of nodes 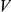 contains random variables and a joint probability distribution 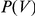 that satisfies the Markov condition with 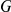. We say that 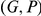 satisfies the *Markov condition* if for each variable 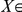 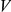, it holds that 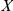 is conditionally independent in *P* of the set of all its nondescendents in 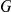 given the set of all its parents in *G*. It is a theorem [14] that 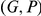 satisfies the Markov condition (and therefore is a BN) if and only if 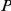 is equal to the product of its conditional distributions of all nodes given their parents in 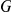, whenever these conditional distributions exist. That is, if our variables are 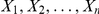, and 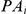 is the set of parents of 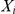, then
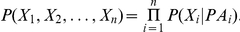
Due to this theorem, BNs are often developed by first defining a DAG that satisfies the Markov condition relative to our belief about the probability distribution of the nodes in the DAG, and then determining the conditional probability distributions for this DAG. Often the DAG is a causal DAG, which is a DAG in which there is an edge from 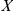 to 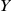 if and only if 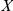 is a direct cause of 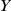 relative to the other nodes in the DAG.

**Figure 1:**
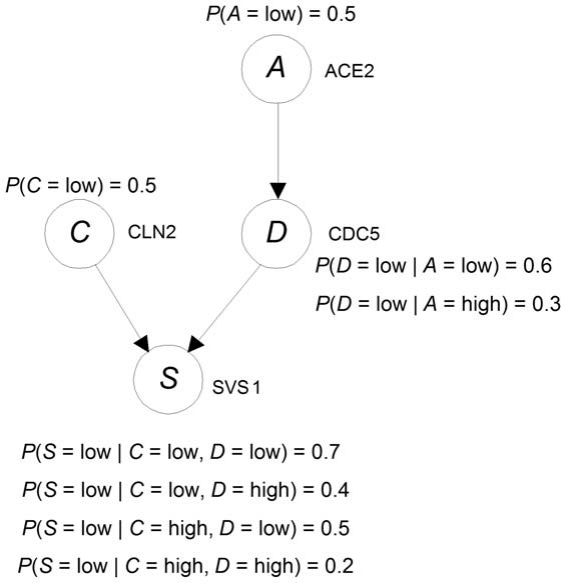
A Bayesian network showing possible relationships among gene expression levels. The levels have been discretized to the values low and high. The network is for illustration purposes only; it is not meant to accurately portray real relationships.

Figure 1 shows a BN representing the causal relationships among gene expression levels. The expression levels have been discretized into two values, 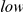 and 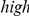. Using this BN, we can determine conditional probabilities of interest using the BN and a BN inference algorithm. For example, if a given individual has 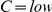 and 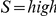, we can for example determine the conditional probability of 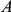 being low and of 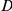 being low. That is, we can compute
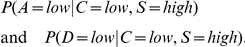



Methods have been developed both for learning the parameters in a BN and the structure (called a *DAG model*) from data. The research discussed here concerns structure learning, which we discuss next. The task of learning a unique DAG model from data is called model selection. As an example, if we had data on a large number of individuals and their expression levels of the genes shown in Figure 1, we might be able to learn the DAG in Figure 1 from data. When the edges represent causal influences, this means we can learn causal influences from data under assumptions. In the score-based structure learning approach, we assign a score to a DAG based on how well the DAG fits the data.

Cooper and Herskovits [30] developed the *Bayesian score* for discrete variables, which is the probability of the 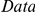 given the DAG. This score uses a Dirichlet distribution to represent our prior belief for each conditional probability distribution in *G* and contains hyperparameters that represent these prior beliefs. The score is as follows:
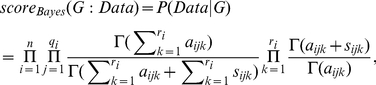
(1)where

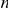
 is the number of variables in the DAG model *G*;

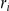
 is the number of states of 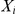;

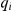
 is the number of different values that the parents of 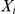 in 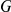 can jointly assume;

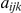
 is our assessed prior belief from previous experience (before obtaining the current data) of the number of times 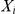 took its 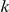
th value when the parents of 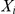 took their
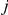
th value;

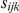
 is the number of times in the data that 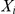 took its 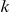
th value when the parents of 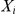 took their 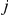
th value.

The Bayesian score does not necessarily assign the same score to Markov equivalent DAG models. Two DAGs are *Markov equivalent* if they entail the same conditional independencies. For example, the DAGs *X*→*Y* and *X*←*Y* are Markov equivalent. Heckerman et al. [31] show that if we determine the values of the hyperparameters from a single parameter 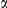 called the *prior equivalent sample size* then Markov equivalent DAGs obtain the same score. If we use a prior equivalent sample size 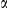 and want to represent a prior uniform distribution for each variable in the network, then for all 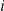, 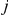, and 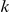 we set 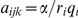, where 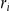 and 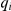 are defined as above. When we use a prior equivalent sample size 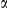 in the Bayesian score, the score is called the *Bayesian Dirichlet equivalent (BDe) score*. When we also represent a prior uniform distribution for each variable, the score is called the *Bayesian Dirichlet equivalent uniform* (*BDeu*) *score* and is given by the following formula, which is a special case of Equation 1:
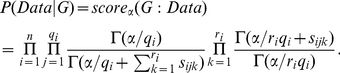
(2)


### Posterior Probabilities of Disease-SNP Models

**Figure 2:**
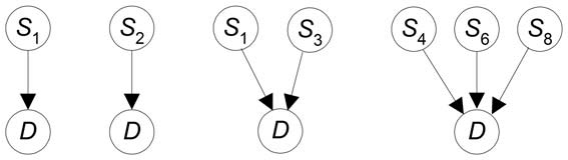
DAG models representing associations between SNPs and a disease.

In what follows, for simplicity we refer to variables that might be associated with a disease as SNPs. However, in general they could be any genetic information or environmental factors. We can represent the relationship between a disease and SNPs using simple DAG models like those shown in Figure 2. The first model represents that SNP 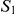 is associated with disease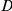. The third model represents that SNPs 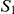 and 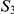 together are associated with 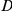 (this could happened because each individually is associated with *D* or because together they are associated with *D* due to an epistatic interaction), and the fourth model represents that SNPs 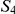, 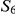, and 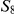 together are associated with 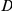.

Our goal is to compute the posterior probability of a model 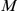 given 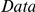. We can do that using Bayes' Theorem as follows:
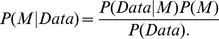
(3)The 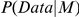 term can be computed using the BDeu score (Equation 2) with a particular choice of 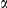. The BDeu score has been used successfully to learn epistatic interactions from real GWAS data sets, and in one analysis [26] it has been shown to more often identify the model generating the data than multifactor-dimensionality-reduction (MDR) [32], a well-known method for learning epistatic interactions. The 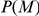 term is the prior probability of 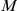. We discuss the assessment of this probability in Supporting Information S1. We call the posterior probability in Equation 3 the *Bayesian Network Posterior Probability* (BNPP). Next we show how to compute the BNPP.

### Computing the BNPP

**Figure 3:**
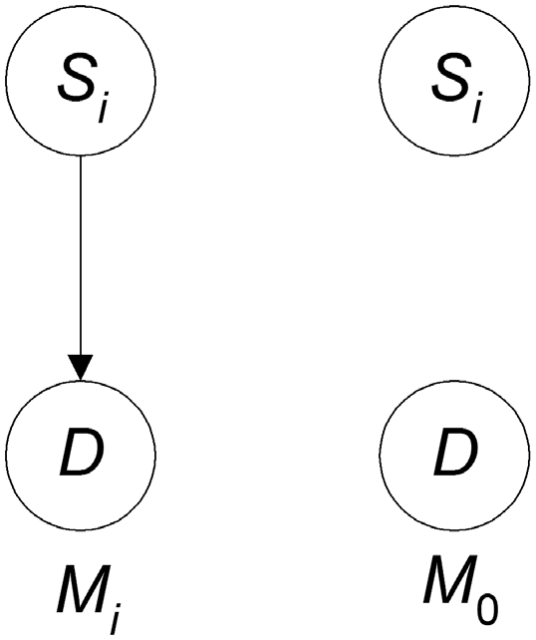
The model that *S_i_* is associated with *D* all by itself is on the left and the model that it is not is on the right.

Consider first a 1-SNP model. Let 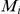 be the model that 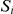 *all by itself* is associated with 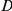 and 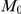 be the model that it is not (see Figure 3). Then the posterior probability of 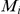 is given by
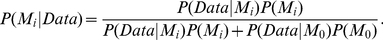
(4)Note that the model in Figure 3 is not just that 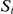 is associated with the disease, but rather that it is associated all by itself. That is, if 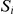 was involved in an epistatic interaction with no marginal effects, the model would be false. Note further that 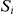 can have any number of discrete values in the model. We are not restricted to only two values as in some of the methods discussed previously. So we can represent all three values of a SNP, or if we are representing an environmental feature with many values we can represent all of them. If the environmental feature is continuous, we can discretize it. So we overcome Difficulty 3 mentioned in the introduction (recall that Difficulty 3 is that the odds ratio only considers two possibilities, either a condition is present or it is not).

**Figure 4:**
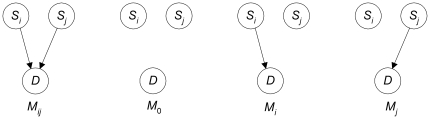
The model that *S_i_* and *S_j_* together are associated with *D* is on left; the three competing models are on the right.

Figure 4 shows the model 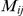 that 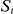 and 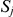 together are associated with 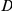 (without needing other interacting SNPs). Note that this model includes the possibility that there is epistasis with no marginal effects, as well as the possibility that each SNP by itself has an association with *D*. The three competing models are on the right. Note further that the model denoted as 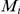 is not the same as the model 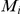 in Figure 3. Model 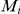 in Figure 4 represents that 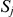 is not associated with 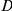 either by itself (other than possibly through 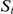) or together with 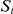, whereas model 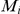 in Figure 3 says nothing about 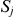.

No other method discussed in the introduction considers these multiple competing hypotheses. They would only consider the null hypothesis 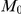 in which no association with 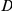 holds versus 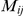. However, if either model 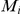 or 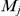 were the correct model, we would observe an association of the two SNPs together with 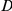 (and therefore reject 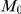) even though 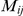 is incorrect. An example of this situation is the relationship between *APOE* and rs41377151, which will be discussed when we analyze an Alzheimer's data set in the Results section. So we attend to Difficulty 4 mentioned in the introduction (recall that Difficulty 4 is that other methods only consider a null hypothesis and an alternative hypothesis).

The posterior probability of 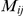 is as follows:
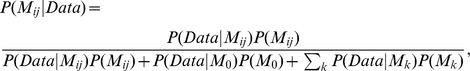
where 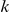 sums over the two 1-SNP models.

**Figure 5:**
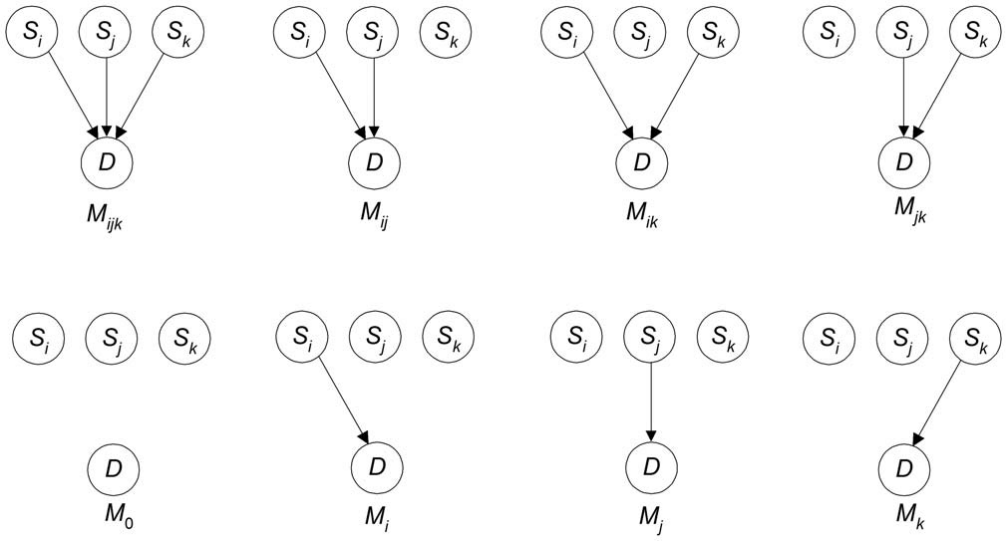
A 3-SNP model and its competing models.

Figure 5 shows a 3-SNP model and the competing models. The number and complexity of the competing models increases with the size of the model. However, we need not identify all the competing models because we have developed the following recursive algorithm for computing 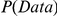 for an arbitrary number of SNPs, which is the denominator in the formula for the posterior probability of a model:

**Algorithm:** Compute 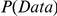.

The SNPs in the model being evaluated are *S*_1_, *S*_2_,…., *S_n_*.


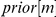
 is the prior probability of an 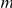-SNP model.

We assume all *m*-SNP models have the same prior probability, but this assumption is not necessary.
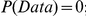



**for** 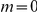 **to** 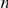


 
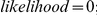



 
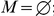



 
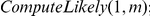



 
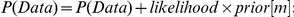



**endfor**

**procedure** 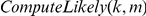 // 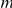 is the size of the model being considered.

**if** 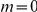


 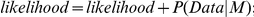 // *likelihood* and *M* are global to this procedure.

**else**

 **for** 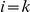 **to** 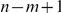


  add *S_i_* **to** 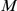;

  
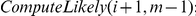



  remove *S_i_* from 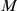;

 **endfor**

**endif**

There are *n* SNPs in the model being analyzed. The algorithm proceeds by calling procedure *Computelikely* for every *m*≤*n*. For each value of *m* this routine then computes the contribution of all *m* SNP models to the likelihood by recursively visiting all such models. Since every subset of the *n* SNPs determines a competing model, the likelihoods for 2*^n^* models are computed. However, since ordinarily there are at most 5 SNPs in a model, this computation is feasible.

There are various possibilities for the data structure we could use in representing a model. We currently choose to represent a model simply as an *n*-element array *M*, where *M*[*i*] contains the index of the *i*th SNP in the model. For example, if *n* = 3 and *S*_2_, *S*_4_, and *S*_10_ are the SNPs in the model, then *M*[1] = 2, *M*[2] = 4, and *M*[3] = 10.

## Results

Next we present results of evaluating the BNPP using both simulated and real data sets. All experiments were done using a Macbook Pro notebook with a 2.66 GHz processor and 8 GB of RAM. For the sake of focus, in what follows we will always refer to the phenotype as a disease.

### Simulated Data

Velez et al. [33] created 70 epistasis models that are described in Supplementary Table one to that paper. Each model represents a probabilistic relationship in which two SNPs together are statistically associated with the disease, but neither SNP is individually predictive of disease. The relationships represent various degrees of penetrance, heritability, and minor allele frequency. Data sets were generated with case-control ratio of 1∶1. To create one data set they fixed the model. Based on the model, they then generated data concerning the two SNPs that were related to the disease in the model, 18 other unrelated SNPs, and the disease. For each of the 70 models, 100 data sets were generated for a total of 7000 data sets. This procedure was followed for data set sizes equal to 200, 400, 800, and 1600. The data sets were generated separately. See http://discovery.dartmouth.edu/epistatic_data to obtain these data sets.

For each of these data sets, we computed the posterior probability of each 1-SNP, 2-SNP, and 3-SNP model using the BNPP, making a total of 1350 models investigated per data set. As discussed in Supporting Information S1, researchers estimate that in an agnostic study the prior probability of an individual SNP being associated with a disease is between 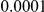 and 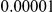. An *agnostic study* is an explorative study in which we have no special prior belief concerning any particular locus. Lower and upper posterior probabilities were obtained using each of these priors for an individual SNP being associated with a disease, and using the strategy for determining model priors based on individual SNP priors, which is also presented in Supporting Information S1. To compute the likelihoods the BDeu score (Equation 2) was used. The hyperparameter 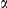 was set equal to 54 because this value yielded good epistasis discovery in a previous study [27] using the Velez and other data.

Table 1 shows the results. The average probability of the true models is much higher than that of the false models. Furthermore, this average probability is moving toward 1 as the sample size increases, whereas that of the false models remains quite small. Finally, the results for the two different priors are not substantially different. This robustness result is encouraging because the assessment of priors is arguably the most onerous part of a Bayesian analysis.

**Table 1:** The posterior probability results for the simulated data sets.

prior probability	sample size	times true model was highest	avg. posterior probability of true models	avg. posterior probability of best false models	avg. posterior probability of all false models
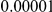	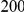	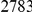	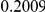	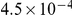	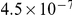
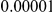	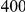	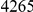	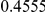	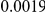	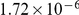
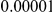	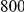	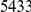	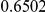	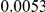	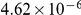
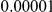	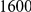	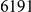	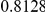	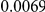	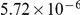
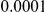	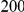	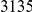	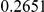	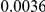	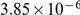
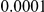	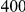	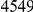	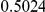	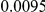	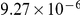
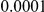	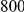	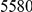	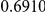	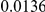	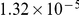
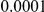	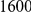	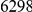	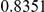	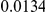	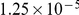

The 1st column shows whether the smaller or larger priors were used; the 3rd column shows the number of times (out of 7000 data sets) the true (i.e., the data-generating) model had the highest posterior probability; the 4th column shows the average posterior probability of the true models; the 5th column shows the average posterior probability of the most probable false models (in each of the 7000 data sets); and the last column shows the average posterior probabilities of all false models.

We repeated the analysis using 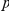-values obtained from Pearson's chi-square test. Table 2 shows the results. These *p*-values are uncorrected since a Bonferroni or Šidák correction would be the same for all of them, and therefore not change the relative order. Notice that the average 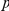-value of the best false models is smaller than that of the true models (recall that smaller *p*-values are more significant). Table 1 shows that the average posterior probability of the best false models is smaller than that of the true models (larger posterior probabilities are more significant).

**Table 2:** The *p*-value results for the simulated data sets.

sample size	avg. *p*-value of true models	avg. *p*-value of best false models	avg. *p*-value of all false models
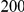	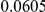	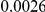	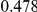
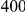	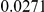	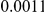	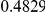
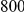	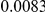	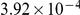	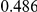
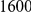	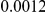	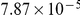	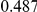

The values are like those in Table 1 except they concern the *p*-values obtained using Pearson's chi-square test.

The performance of an evaluation method can be judged by how high it ranks true models and how low it ranks false models. The previous results support that the BNPP algorithm exhibits better evaluation performance than the method based on *p*-values.

**Figure 6:**
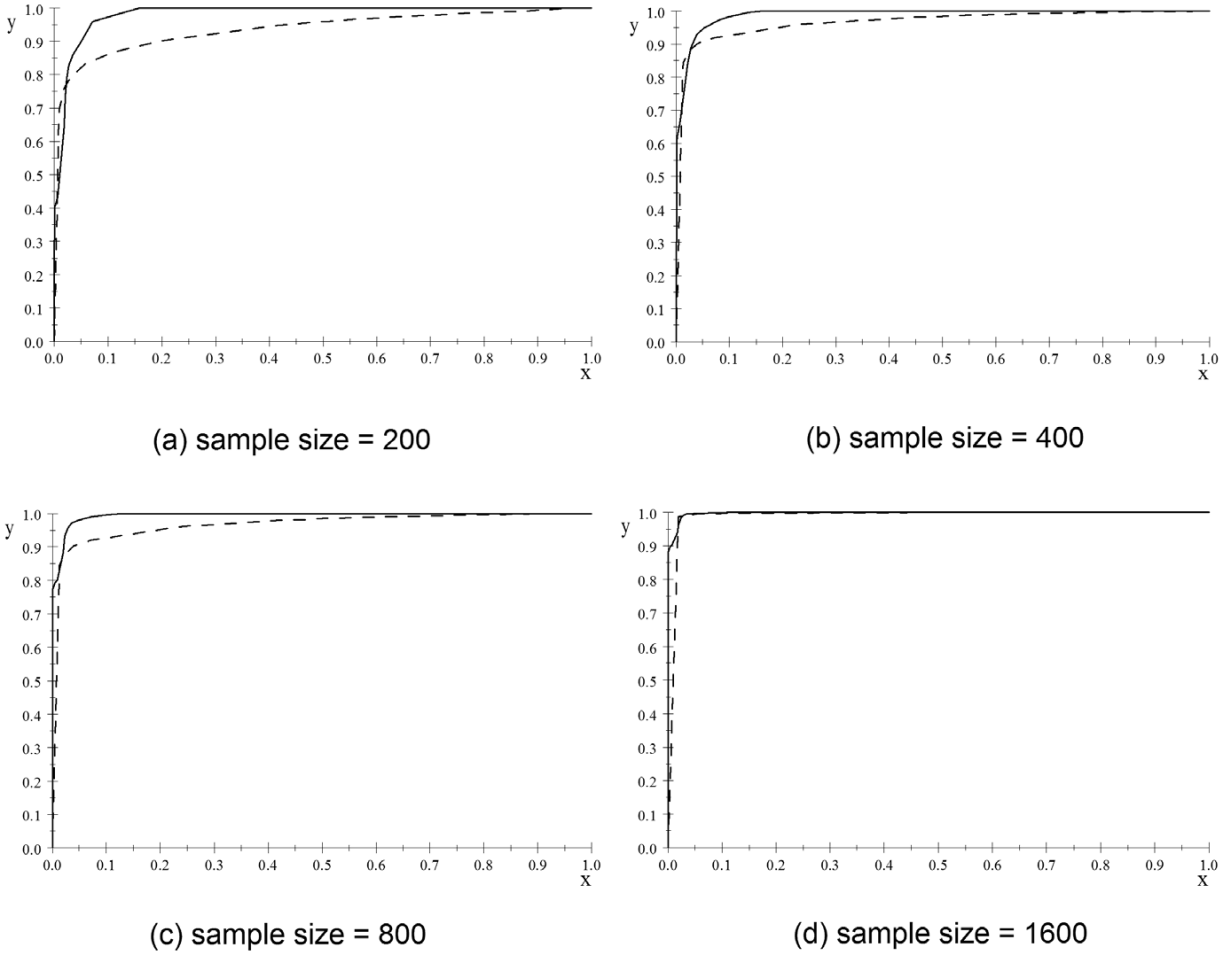
ROC curves concerning the posterior probabilities when the prior is 0.00001 and the *p*-values for the simulated data sets. The curve for the posterior probability is a solid line, while the one for the *p*-values is a dashed line. 1-specificity is on the *x*-axis and the sensitivity is on the *y*-axis.

Next we address discovery. Figure 6 shows ROC curves concerning the posterior probabilities when the individual SNP prior is 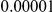 and the 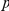-values for the simulated data sets. The results for the posterior probabilities were almost identical when the prior was 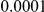; so we do not show them. A *receiver operating characteristic* (ROC) curve plots the true positive rate (sensitivity) on the 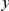-axis and the false positive rate (1 - specificity) on the 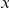-axis. It is obtained by considering various threshold probabilities as being binary indicators of discovery. For example, the point 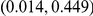 appears on the curve for the posterior probability in Figure 6 (a) because 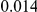 fraction of the false models have posterior probabilities exceeding a threshold (in this case 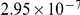), while 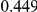 fraction of the true models have posterior probabilities exceeding this threshold. The point 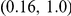 appears on the curves in Figures 6 (a), (b), and (c) and the point 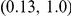 appears on the curve in Figure 6 (d). This means that if we were using the posterior probability as a binary indicator of discovery in the case of samples sizes of 200, 400, or 800, we could discover all the true models with a false discovery rate of about 16% (based on this analysis). On the other hand, the true positive rate for the 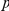-value does not reach 1 until the false positive rate reaches 1. This is true even when the data set has size 1600 (this is not noticeable in the display of its ROC curve). This result supports the effective discovery performance of the BNPP.

**Figure 7:**
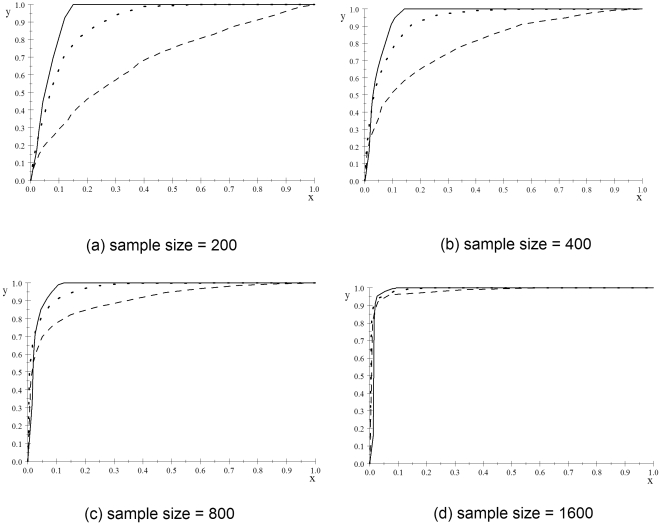
ROC curve concerning the posterior probabilities when the prior is 0.00001 and the *p*-values for models 55–59. The curve for the posterior probability is a solid line, the one for the *p*-value is a dashed line, and the one for the *p*-value with the Šidák correction is a dotted line. 1-specificity is on the *x*-axis and the sensitivity is on the *y*-axis.

Velez et al. [33] showed that models 55–59 in the Velez Data are the most difficult models to learn. They have the weakest broad-sense heritability (0.01) and a minor allele frequency of 0.2. These models are arguably most like relationships we might find in nature. ROC curves concerning only these models appear in Figure 7. Although the curves for the posterior probability are not that much worse than when we consider all models, the ones for the 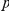-value are substantially worse except when the sample size is 1600. The worst possible ROC curve is a straight line from (0,0) to (1,1). The 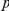-value ROC curve when the sample size is 200 is not much better than that line.

We can perhaps apply different corrections to different sized models and stay in the framework in which the correction is applied by arguing that 1-SNP models, 2-SNP models, and 3-SNP models are different families of models and we should apply different corrections for each of these families. Since there were 20 SNPs total in the simulations, we applied the Šidák correction using 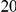 for 1-SNP models, 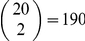 for 2-SNP models, and 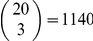 for 3-SNP models. The resultant curves appear with a dotted line in Figure 7. Although we have improved the results, they are still not as good as those for the posterior probability. Also, if we did a study with a different number of SNPs, we would need to apply a different correction, and we would expect to obtain different results. On the other hand, the BNPP model suggests using the same prior probabilities across all agnostic studies.

The average times to compute the posterior probabilities and the *p*-values for all one to three SNP models were 1.8 seconds and 0.7 seconds respectively.

### Real Data

#### Alzheimer's Data set

Reiman et al. [3] analyzed a GWAS late onset Alzheimer's disease (LOAD) data set on 312,317 SNPs from an Affymetrix 500K chip, plus the measurement of a locus in the *APOE* gene, which is known to be predictive of LOAD. The data set consists of three cohorts containing a total of 1411 participants. Of the 1411 participants, 861 had LOAD and 550 did not. In addition, 644 participants were *APOE* 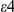
carriers, who carry at least one copy of the *APOE* 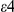 genotype and 767 were *APOE* 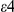 non-carriers. See http://www.tgen.org/neurogenomics/data concerning this data set. Reiman et al. found the *APOE* gene is significantly associated with LOAD, the *GAB2* gene is not significantly associated with LOAD, the *GAB2* gene is significantly associated with LOAD in *APOE* 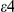 carriers, and the *GAB2* gene is not significantly associated with LOAD in the *APOE* 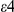 non-carriers. These results indicate that *APOE* and *GAB2* may interact epistatically to affect LOAD. Using these same data, we computed the posterior probability of each locus being associated with LOAD (1-locus models), and the posterior probability of each locus together with *APOE* being associated with LOAD (2-locus models).

**Figure 8:**
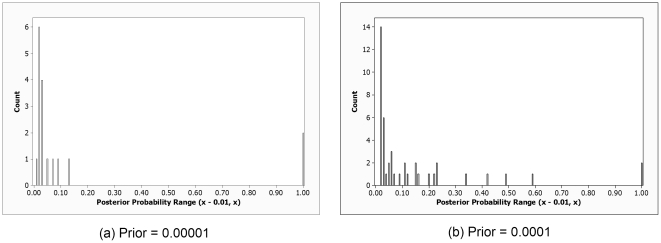
Bar charts showing the number of 1-locus models in each posterior probability range. The posterior probability is that of the model in which a single locus is associated with LOAD.

The average posterior probability of all 1-locus models was 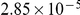 for the individual SNP prior equal to 0.0001, and 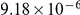 for that prior equal to 0.00001. Furthermore, the numbers of models (loci) with posterior probabilities less than 0.01 were respectively 312,301 and 312,273 for the two priors. Figure 8 shows bar charts depicting the results concerning the remaining loci. Table 3 shows the loci in the 10 most probable models. *APOE* has a posterior probability of ∼1, regardless of the prior, as does SNP rs41377151. SNP rs41377151 is on the *APOC1* gene, which is in strong linkage disequilibrium with *APOE* and for which previous studies have indicated that they predict LOAD equally well [34]. The 3rd most probable locus is rs1082430, which is on the *PRKG1* gene. There are a number of previous studies associating this gene with LOAD [35], [36]. Of the seven remaining probable loci, there is some previous evidence linking four of them to LOAD [37].

**Table 3:** Results concerning the 10 most probable 1-locus models in the LOAD study in [3].

locus	posterior probability range	previous LOAD association
*APOE*	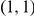	Yes
rs41377151	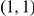	Yes
rs10824310	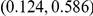	Yes
rs4356530	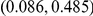	No
rs17330779	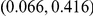	Yes
rs6784615	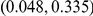	Yes
rs10115381	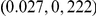	No
rs12162084	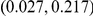	Yes
rs4862146	(0.024,0.192)	No
rs249153	(0.017,0.152)	Yes

As mentioned in Supporting Information S1, as more genome-wide association studies are carried out we will better be able to assess appropriate priors. These results indicate that 0.00001 may be more appropriate than 0.0001 since the latter prior resulted in fairly high posterior probabilities for three SNPs that have no known previous association with LOAD; nonetheless, these might be valid predictors of LOAD that have not been appreciated previously.

**Figure 9:**
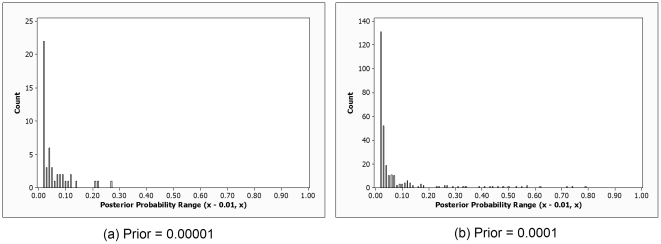
Bar charts showing the number of models in each posterior probability range. The posterior probability is that of the 2-locus model in which each locus together with *APOE* is associated with LOAD.

The average posterior probability of all 2-locus models, in which one of the loci was *APOE*, was 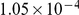 for the individual SNP prior equal to 0.0001 and 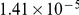 for that prior equal to 0.00001. Furthermore, the numbers of models with posterior probabilities less than 0.01 were respectively 312,267 and 312,028 for the two priors. Figure 9 shows bar charts depicting the results concerning the remaining models. Table 4 shows the loci in the ten most probable models. Eight of those loci are SNPs located on the *GAB2* gene. The prior probability of a 2-SNP model is 6×10^−10^ when the individual SNP prior is 0.0001 and 6×10^−12^ when that prior is 0.00001 (See Supporting Information S1). We see from Table 4 that the posterior probabilities of 2-locus models containing APOE and a GAB2 SNP are much greater than these prior probabilities. On the other hand, the 1-locus models containing *GAB2* SNPs had posterior probabilities about equal to their prior probabilities. These results together indicate *GAB2* by itself does not affect LOAD, but that *GAB2* interacts with *APOE* to affect LOAD.

**Table 4:** Results concerning the ten most probable 2-locus models, where one locus is *APOE*, in the LOAD study in [3].

locus	posterior probability range	*GAB2*
rs1007837	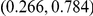	Yes
rs7101429	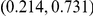	Yes
rs901104	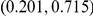	Yes
rs4291702	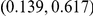	Yes
rs4945261	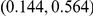	Yes
rs12162084	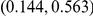	No
rs7115850	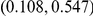	Yes
rs10793294	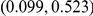	Yes
rs2450130	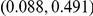	Yes
rs6784615	(0.081, 0.462)	No

The two loci in the top ten 2-locus models that are not on *GAB2*, namely SNPs rs6784615 and rs12162084, are among the 10 most probable 1-locus models (see Table 3). These results together indicate that each of these SNPs may affect LOAD independently of *APOE*. As indicated in Table 3, previous studies have associated these SNPs with LOAD.

Another interesting result is that *APOE* and rs41377151 (the two loci with posterior probabilities about equal to 1 in Table 3), when considered together, had posterior probabilities of 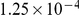 and 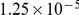 for the individual SNP priors of 0.0001 and 0.00001 respectively. This result indicates that the model containing both loci is incorrect. As mentioned above, SNP rs41377151 is on the APOC1 gene, and previous investigations have shown that APOE and APOC1 are in linkage disequilibrium and each of them predicts LOAD as well as the other [34]. However, we know of no previous study substantiating that the two loci identify the same single causal mechanism of LOAD. This result could not have been obtained with a method that only considered the null hypothesis that the two loci together are not associated with LOAD, and the alternative hypothesis that they are. For example, using Pearson's chi-square test, we obtained *p*-values all equal to ∼0 for *APOE* alone, rs41377151 alone, and *APOE* and rs41377151 together (the 2-locus model). The BNPP determined that the 2-locus model is improbable because it also evaluated the competing hypotheses that only one locus is directly causative of LOAD. To learn that the 2-locus model is not significantly better than the 1-locus model using commonly applied frequentist statistics, we would need to perform an analysis such as stepwise regression or regression on the two loci followed by an investigation of the coefficients.

The three interesting results just discussed (the first concerning *GAB2*, the second rs6784615 and rs12162084, and the third rs41377151) follow from our computing the posterior probabilities of all 1-locus models and all 2-locus models containing *APOE*. It was not necessary to suspect any of them ahead of time or perform a focused analysis.

The running times were 196 seconds and 193 seconds to investigate all 312,318 1-locus models using individual SNP priors 0.0001 and 0.00001, respectively. The corresponding running times to investigate all 2-locus models containing *APOE* were 593 seconds and 584 seconds.

#### Breast Cancer Data set

Hunter et al. [1] conducted a GWAS concerning 546,646 SNPs and breast cancer as part of the National Cancer Institute *Cancer Genetic Markers of Susceptibility* (CGEMS) Project. (see http://cgems.cancer.gov/.) They determined the significance of each SNP using logistic regression with two degrees of freedom. Two of the six most significant SNPs were on the *FGFR2* gene. Furthermore, two other *FGFR2* SNPs were among the 16 most significant SNPs. Previously, it was known that *FGFR2* is amplified and overexpressed in breast cancer [38], [39]. Furthermore, a large, three-stage GWAS of breast cancer had identified SNPs in *FGFR2* as the strongest of its associations [2]. Based on their results and these previous findings, Hunter et al. [1] investigated *FGFR2* in three additional studies and found further support for an association of *FGFR2* with breast cancer.

Using this same GWAS data set, we computed the posterior probability of all 1-locus models using the agnostic individual SNP priors of 0.00001 and 0.0001 and the informative priors of 0.01 and 0.1. The average posterior probability of all 1-locus models was 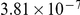 for the prior equal to 0.00001 and 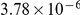 for the prior equal to 0.0001. Furthermore, the numbers of models (loci) with posterior probabilities less than 0.01 were respectively 546,645 and 546,637 for the two priors. Table 5 shows results concerning the ten most probable models. Columns 2–5 show posterior probabilities while Columns 6 and 7 show *p*-values and Sidák-corrected *p*-values. The six most significant SNPs discovered by Hunter [1] are in our ten most probable models. These are the SNPs for which we show *p*-values, which were obtained from [1]. However, we performed the Sidák-correction as this was not done in [1].

**Table 5:** Results concerning the ten most probable models in the breast cancer study in [1].

SNP	prior = 0.00001	prior = 0.0001	prior = 0.01	prior = 0.1	*p*-value	Šidák	previous BC association
rs10510126	0.0118	0.1185	0.9967	0.9992	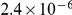	0.7307	No
rs17157903	0.0031	0.0306	0.9693	0.9968	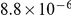	0.9919	Yes
rs2420946 (*FGFR2*)	0.0022	0.0218	0.9570	0.9955	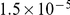	0.9997	Yes
rs1219648 (*FGFR2*)	0.0021	0.0209	0.9552	0.9953	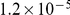	0.9986	Yes
rs7696175	0.0013	0.0131	0.9298	0.9925	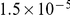	0.9997	Yes
rs197275	0.0012	0.0123	0.9256	0.9920	Not Avl.	Not Avl.	No
rs12505080	0.0012	0.0123	0.9255	0.9920	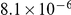	0.9881	No
rs210739	0.0011	0.0114	0.9204	0.9914	Not Avl.	Not Avl.	Yes
rs10779967	0.0011	0.0113	0.9194	0.9913	Not Avl.	Not Avl.	No
rs2981579 (*FGFR2*)	0.0008	0.0083	0.8933	0.9882	Not Avl.	Not Avl.	Yes

Columns 2–5 show posterior probabilities for various priors, while Columns 6 and 7 shows *p*-values (obtained from [1]) and Sidák-corrected *p*-values.

If we consider a result significant based on the Šidák correction, no result would be close to significant and the findings in this study would not support any of the SNPs being predictive of breast cancer. Given the considerable prior knowledge concerning *FGFR2*, we can follow a practice established in Wacholder et al. [12] of assigning a prior probability of 0.01 to 0.1 to an *FGFR2* SNP. Using even the smaller of these priors, our Bayesian analysis of these data strongly supports that *FGFR2* is associated with breast cancer. Hunter et al. [1] drew a similar conclusion without performing a formal analysis involving priors. We had no prior belief that SNP rs10510126 was associated with breast cancer, and Hunter et al. [1] did not discuss this SNP further, even though it had the smallest 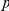-value. However based on our priors for an agnostic search, the posterior probability of this SNP is between 0.0118 and 0.1185, and is much larger than any of the other posterior probabilities. Based on this result and the utility of further analysis (see the Conclusions section), this SNP appears to warrant additional study.

Besides the three *FGFR2* SNPs, three other SNPs in the top ten have been previously associated with breast cancer [40], [41]. See Table 5.

#### A Comparison to the FPRP

Kuschel et al. [42] investigated 16 SNPs in seven genes involved in the repair of double-stranded DNA breaks and breast cancer in a case-control study involving 2200 cases and 1900 control subjects. Using standard significance testing, they found two polymorphisms in *XRCC3* and one polymorphism in each of *XRCC2* and *LIGA* to be the most significant. They also performed a haplotype analysis investigating the effect of the genetic variants in the *XRCC3* gene on breast cancer. Wacholder et al. [12] analyzed these same data using the FPRP method. Statistical power in their analysis was the power to detect an odds ratio of 1.5 for the homozygote with the rare genetic variant and an odds ratio of 1.0 for the homozygote with the common variant. Based on previous findings [40], [41], Wacholder et al. [12] assigned a prior range of 0.01 to 0.1.

We analyzed these same data using the BNPP algorithm to obtain the posterior probabilities of the models. Table 6 shows the results. The last two columns show posterior probabilities of association with breast cancer; to make comparisons easier, we show 1-FPRP in columns 3 and 4. The 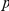-values in the second column were computed using the chi-square test with two degrees of freedom.

**Table 6:** *p*-values, FPRP values, and BNPP values for five results concerning association with breast cancer in the study in [42].

Gene/SNP	*p* - value	1-FPRP prior = 0.01	1-FPRP prior = 0.1	BNPP prior = 0.01	BNPP prior = 0.1
*XRCC3* 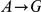 at nt 17893	0.008	0.570	0.936	0.1186	0.5967
*XRCC3* 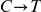 at nt 18067	0.015	0.410	0.880	0.0539	0.3854
*XRCC2* 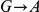 at nt 31479	0.070	0.020	0.210	0.0145	0.1419
*LIG4* 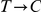 at nt 1977	0.090	0.090	0.520	0.0277	0.2384
*XRCC3* haplotype	.000016	0.9984	0.99985	0.9749	0.9983

The FPRP and BNPP exhibit similar results concerning the four SNPs and the haplotype, however, the results for BNPP are more conservative. Recall that a particular value of the odds ratio (1.5) was used for statistical power in the case of the FPRP. A larger value would result in smaller posterior probabilities. The BNPP makes no assumptions about a statistic such as the odds ratio; it only conditions on the models being true. Note that the posterior probabilities (using both the FPRP and BNPP) for the *LIG4* SNP are somewhat larger than those for *XRCC2* SNP even though the latter SNP has a smaller 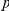-value. Wacholder et al. [12] discuss how this result may be due to the fact that there is very little data concerning the rare homozygote in the case of the *XRCC2* SNP.

### A Decision Analytic Approach to Using the BNPP

The question remains as to what to with BNPP results. In an agnostic GWAS investigation the prior probabilities are ordinarily very low. So, given the limited number of samples in current GWAS data sets, often the posterior probabilities of even our most probable models are not very high. For example, consider the result in Table 5 that the posterior probability of rs10510126 being associated with breast cancer is either 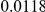 or 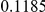 depending on whether the prior probability is 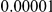 or 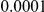. The average of these values, namely 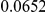, can be used to represent our posterior belief in the validity of this association. This value is not very high, and so one may ask whether it is significant. In general, statistics cannot tell us whether a result is significant; it can only change our belief. It has become a controversial practice by some to consider a *p*-value of 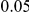 or smaller to be significant largely because of R.A. Fisher's [43] statement in 1926 that “it is convenient to draw the line at about the level at which we can say: Either there is something in the treatment, or a coincidence has occurred such as does not occur more than once in twenty trials.” However, as has been often discussed, there is nothing special about the value 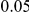 for a 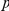-value that enables a dichotomous announcement, just as there is nothing special about a particular posterior probability.

The value 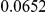 represents our belief concerning the truth of the model based on our knowledge concerning the model, namely our prior belief and the data. Although we cannot dichotomously announce whether the value is significant, we can use it to make a decision about what to do. We should report the finding concerning model *M* if the expected utility of not reporting *M* is less than the expected utility of reporting *M*. Let *U_TD_* be the utility of a true discovery, which is the utility of reporting a true model, *U_FD_* be the utility of a false discovery, which is the utility of reporting a false model (and which is therefore negative), *U_TND_* be the utility of a true non-discovery, which is the utility of not reporting a false model, and *U_FND_* be the utility of a false non-discovery, which is the utility of not reporting a true model (and which is therefore negative). We should report model *M* if
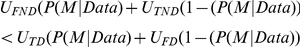
or
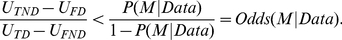
(5)In the current analysis, 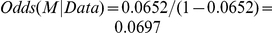. So we should report the finding (and therefore investigate the model further) if 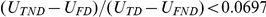.

If we take this decision-analytic approach to using the BNPP, we conclude that it provides researchers with a useful tool for guiding how they should proceed based on their findings.

Wakefield [13] proposed a formula similar to Equation (5), but only considered the *U_FD_* and the *U_FND_*. That is, the utilities of a true discovery and of a true non-discovery were not factored into the decision.

## Discussion

We identified four difficulties with many current methods for computing the posterior probability of a model analyzed using a GWAS data set. Most importantly, they only consider a null hypothesis 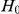 and an alternative hypothesis 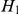. So, they cannot handle a complex multi-locus hypothesis which has several competing hypotheses. Yet it is becoming increasingly commonplace to investigate multi-locus hypotheses. We developed the BNPP method which enables us to compute the posterior probability of such hypotheses, and which also attends to the other difficulties. We illustrated its effectiveness by applying it to both simulated and real data sets. We showed how the BNPP can be used to obtain a decision analytic solution as to when to report a finding.

The greatest difficulty in most Bayesian analyses is arguably the assessment of prior probabilities. The early rejection and now the slow acceptance of the Bayesian approach has been due in large part to the perceived arbitrary nature of these assessments. For example, in 1921 R.A. Fisher [44] stated that “The Bayesian approach depends upon an arbitrary assumption, so the whole method has been widely discredited.” However, the Bayesian approach does provide an elegant and general solution to the multiple hypothesis testing problem. Let 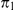 denote the prior probability that the model is correct. Wakefield [13] points out that “as more genome-wide association studies are carried out lower bounds on 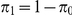 will be obtained from the confirmed ‘hits’ - it is a lower bound since clearly many non-null SNPs for which we have low power of detection will be missed.” We agree that in time results will help us to determine priors. However, one avenue of research that builds on the results presented here, would be to hasten this process by performing a comprehensive literature search to investigate current beliefs concerning agnostic priors. Supporting Information S1 proposes initial prior probabilities based on beliefs reported in two articles.

The BNPP was designed for the purpose of *flagging SNPs for further investigation*; that is, it is intended to compute the posterior probability of a model that was already discovered or conjectured. However, as mentioned at the beginning of the Methods section, the BNPP is also a promising technique for loci-disease association discovery. Indeed, in order to illustrate the effectiveness of the BNPP we showed results in which it was used for discovery. Future research can further investigate its discovery capability and compare its performance to other related discovery methods such as those appearing in [10], [24], [26]–[28].

An immediate plan we have for using the BNPP is the following. We will expand our previous work on discovery to develop a system that outputs likely one to five SNP models in the first stage. The BNPP will then be used in the second stage to compute the posterior probability of each model consisting of a subset of the SNPs from each of these models. The most probable models will be reported. This method will be compared to using the BNPP directly for discovery.

## Supporting Information

Supporting Information S1 Assessing Prior Probabilities.
(DOC)
