# Supplementary material for: A Bayesian Method for Evaluating and Discovering Disease Loci Associations
Source: PLoS One. 2011 Aug 10;6(8):e22075. doi: 10.1371/journal.pone.0022075 (PMC3154195; doi:10.1371/journal.pone.0022075)
Supplement: Supporting Information S1 — Assessing Prior Probabilities. (DOC) [file pone.0022075.s001.doc]

**Supporting Information S1**

**Assessing Prior Probabilities**

As mentioned in the Discussion section, the biggest challenge in many Bayesian analyses is the assessment of prior probabilities. This appendix presents one method for doing so. The agnostic priors assessed here are the ones that were used in the studies reported in the Results section.

Researchers in the Wellcome Trust Case Control Consortium [23] assessed that there are 1,000,000 regions of correlated SNPs in the genome and an expectation of 10 regions having an effect on phenotype. They therefore assign a prior probability of 0.00001 that a SNP is associated with a given disease. However, they note that other plausible estimates may vary from this prior probability by an order of magnitude or so in either direction. Using similar assumptions, Wacholder et al. [12] arrive at a prior probability between 0.0001 and 0.00001 that a randomly select nonsynonomous variant is associated with a complex disease. Based on these analyses, in an agnostic search we will assume that each individual SNP has between a 0.0001 and a 0.00001 prior probability of being associated with a given disease. The following discussion assumes that there are 1,000,000 regions of correlated SNPs, 10 regions have an effect on phenotype, and so any particular SNP has a prior probability of 0.00001 of affecting phenotype. The calculations can be repeated using a value of 0.0001. We assume that the individual SNP prior probability of 0.00001 is equally distributed among the possibilities of it exhibiting an association all by itself, of it requiring one other SNP to exhibit an association, of it requiring two other SNPs to exhibit an association, and so on up to 10 possible SNPs. So we assume that the prior probability of each of these events is . When we say that the SNP requires one other SNP to exhibit an association, we mean that the SNP shows no association by itself, and there is at least one other region with which it does show an association. We then have the following:

1. Prior probability for a 1-SNP model $M_{i}$ (Note that this applies to the models in Figures 7, 8, and 9 because the prior probability of a SNP not having an edge is almost 1):
2. Prior probability of a 2-SNP model $M_{\text{ij}}$:

The far right term is obtained as follows: $S_{j}$ comes from one of the $1, 000, 000$ assumed regions. There are $999, 999$ remaining regions. We assume that 9 of these regions are associated with the phenotype, and that it is equally likely that $S_{j}$ shows a 2-SNP effect with only one of them, with two of them, and so on up to all nine of them. We then have that the probability of $S_{j}$ showing an effect with $S_{i}$ (given $S_{j}$ does show a 2-SNP effect) is as follows:

1. Prior probability of a 3-SNP model $M_{\text{ijk}}$:
2. Prior probability of a 4-SNP model $M_{\text{ijkl}}$:

We repeat these calculations using $0.0001$ as the prior probability of a SNP being associated with a disease. In this way we obtain upper and lower priors.
